# ‘Does levothyroxine improve pregnancy outcomes in euthyroid women with thyroid autoimmunity undergoing assisted reproductive technology?’

**DOI:** 10.1186/s13044-018-0052-y

**Published:** 2018-05-30

**Authors:** Kris Poppe, Flora Veltri, Candice Autin

**Affiliations:** 1 Endocrine Unit Centre Hospitalier Universitaire Saint Pierre, Rue Haute 322, 1000 Brussels, Belgium; 2 Departement of Gynecology, Obstetrics, and Reproductive Medicine Unit Centre Hospitalier Universitaire Saint Pierre, Rue Haute 322, 1000 Brussels, Belgium; 3 Université Libre de Bruxelles (ULB), 1050 Brussels, Belgium

**Keywords:** ART, ICSI, IVF, Infertility, Miscarriage, Pregnancy outcome, Thyroid function and autoimmunity

## Abstract

Evidence on the treatment of euthyroid infertile women and thyroid autoimmunity with thyroid hormone is scare, and therefore the recent study by Wang et al. is a welcome addition to it. Based on their results, thyroid hormone seems not to be indicated to decrease the miscarriage rate in that particular group of infertile women. This comment is meant to put the study results into perspective with the available evidence and the current guidelines, and to highlight its strengths and weaknesses.

## Background

Thyroid autoimmunity (TAI), defined by the presence of increased levels of anti-thyroid peroxidase antibodies (TPO-abs) and/or anti-thyroglobulin antibodies (Tg-abs) is a frequent finding in the general population (~ 10% depending on the iodine intake of the investigated population), and is even higher in infertile women with polycystic ovaries and idiopathic infertility (~ 20–30%) [1, 2]. Women with TAI and treated with ovarian hyperstimulation as preparation of an assisted reproductive technology (ART) have a more important (and often definitive) decrease in their thyroid hormone levels compared with women without TAI [3]. Finally, the presence of TAI is associated with an increased risk of a first trimester miscarriage, and a number of reasons for that association have been proposed [4, 5]. Compared with women without TAI, women with TAI are older, have more often concomitant autoimmune disorders (reflected by the presence of anticardiolipin, antinuclear, or lupus anticoagulants), may have an immune imbalance of which thyroid antibodies are markers, and finally do they have mild thyroid hypofunction that subsequently creates a less favourable environment, impairing the in-vitro embryo development and/or the early phase of implantation [6]. Taking into consideration the latter hypothesis, a research question is whether thyroid hormone treatment (LT4) could overcome this relative thyroid dysfunction caused by TAI?

## Main text

In this randomized clinical trial, treatment with LT4 in euthyroid infertile women with TAI and treated with ART (IVF (in-vitro fertilization) or ICSI (intra-cytoplasmic sperm injection)) did not reduce the miscarriage rate before 28 weeks’ gestation compared with placebo (10.3% versus 10.6%) [7].

Strengths of the study were the large number of patients included (all with TPO-abs), and the exclusion of women with other auto-immune antibodies, such as anticardiolipin, antinuclear and lupus anticoagulants. Women in both study groups received the same ovarian stimulation protocols, and the number of (fresh) embryos transferred was comparable. Finally, the initiation of LT4 before the ovarian stimulation procedure (i.e. before pregnancy) and the titration of the LT4 dosage during pregnancy to obtain TSH levels within trimester-specific reference ranges were additional strengths and an amelioration compared with previous studies in the field [7].

Before the study by Wang et al., evidence on LT4 treatment in infertile women with TAI was limited to a few studies with negative outcomes, however, these studies were subject to a number of important limitations [8, 9]. In the study by Negro et al., a non-significant difference in the miscarriage rate was noted (in favour of the LT4 group), but it was underpowered and subject to a type II statistical error [8]. The study by Revelli et al. was retrospective, and three different types of medications were given (LT4, acetyl-salicylic acid, and prednisolone) in a non-randomised way, and only a small number of patients were included [9]. Based on those two studies, the American Thyroid Association (ATA) guidelines on the management of thyroid disorders during pregnancy found the evidence insufficient to determine whether LT4 therapy improves the success of pregnancy following ART in euthyroid women with TAI, however, it is mentioned that 25–50 μg LT4 may be considered given its potential benefits compared to its minimal risk [10].

The results of the study by Wang et al. might weaken this ATA recommendation, but “let’s not throw the (LT4) baby out with the bathwater”. Before concluding that LT4 is not effective to reduce miscarriage rates in euthyroid women with TAI, we believe the study results should be put in perspective with the current evidence and thoroughly analysed.

The incidence of miscarriage in clinically pregnancies (< 20 gestational weeks) is 8–20%, and in case of the presence of TAI, it is up to 30% (4). Taking this into consideration, ~ 10% miscarriage rate in this study reduces its power.

A few years ago in a meta-analysis on the association between TAI and pregnancy outcomes after ART, an increased risk for a first trimester miscarriage was observed compared with that in women without TAI [4]. The odds ratio (OR) in that study was 3.15, while in a recent meta-analysis on the same association, the OR was only 1.44 [5]. Two important reasons can be put forward for that decreasing OR, and at the same time, they might explain (partially) the negative results in the study by Wang et al. A first reason can be the increased use of ICSI as type of ART, since ICSI can bias the impeding effects of thyroid antibodies in the follicular fluid on the fertilization of the egg, and therefore, be the more accurate type of ART in women with TAI [11]. Indeed, in a recent meta-analysis, infertile women with TAI and all treated with ICSI (but not with LT4), had comparable miscarriage (and live birth) rates as women without TAI [12]. In the study by Wang et al. the prevalence of women treated with ICSI was ~ 45% in both study groups. Another important point is the changed definition of a normal thyroid function and subclinical hypothyroidism (SCH) over the years. In the studies performed before 2010, a normal thyroid function during the first trimester of pregnancy was defined as a serum TSH level within the upper limit of the assay (often < 4 to 5 mIU/L), while for the period 2010–2016, it was < 2.5 mIU/L [10, 13]. The lower upper limit in the more recent studies might be a reason why the presence of TAI was not anymore associated with altered ART outcomes, as it was shown in a number of recent studies [14–16].

ICSI and LT4 treatments can also be complementary and improve pregnancy outcomes; ICSI on the in-vitro outcomes, and LT4 by preserving a normal thyroid function during pregnancy. Therefore, it make sense that the negative results in the observational studies on the impact of TAI in infertile women with TSH levels within pregnancy-specific reference range (usually ~ 4.0–4.5 mIU/L) and mainly treated with ICSI are in some way predictive for negative study results in interventional trials, and more in particular in that by Wang et al. [7].

In their discussion, the authors also mention that in a pooled analysis of 3 randomized clinical trials, LT4 treatment on pregnancy outcomes among women with TAI and a normal thyroid function significantly decreased the miscarriage rate (RR:0.45; 95% CI, 0.24–0.82) [17]. However, in two of the three studies in that meta-analysis (> 60% of all women included), serum TSH levels were > 4.0 to 4.5 mIU/L and thus compatible with SCH and not a normal thyroid function. In study by Wang et al., normal thyroid function was defined as a serum TSH < 4.7 mIU/L. Indeed, in the 2017 ATA guidelines, LT4 treatment for women with TPO-abs and a serum TSH above pregnancy-specific reference range (or > 4.0 mU/L if unavailable) is strongly recommended [10].

In most studies, the diagnosis of TAI is based on the presence of TPO-abs only [10]. However, in a prospective study determining the presence of TAI in infertile women, only positivity for Tg-abs was more frequent compared with that of TPO-abs [18]. In the same study, women with only Tg-abs also had higher mean serum TSH levels compared with women with only TPO-abs, and in a recent study in China, women with Tg-abs had higher miscarriage rates compared with women with TPO-abs [19]. Not testing for Tg-abs may therefore lead to misclassification, modify study conclusions and impair the management of pregnant women. The importance of Tg-abs is now also noticed in the ATA guidelines on the management of thyroid disorders during pregnancy [10]. Concerning the currently used cut-off for TPO-ab positivity, a recent study highlights that it may be too high. The authors found a dose-dependent relationship between TPO-ab levels and TSH as well as the risk of premature delivery, and therefore, the use of a population-based, pregnancy-specific, functional cut-off for TPO-abs may be useful in the future [20].

## Conclusions

Before recommending against LT4 treatment in infertile women with TAI and serum TSH levels within pregnancy-specific reference range, we believe that more studies are needed investigating the impact of thyroid function/TAI and ICSI on the in-vitro data (oocyte quality, fertilization rate, embryo quality), the optimal LT4 dosage, and finally, optimising the diagnosis of TAI e.g. by taking Tg-abs into account as an equal marker as positivity for TPO-abs. In the meantime, physicians should judge case by case whether LT4 should be given, taking into account other parameters such as baseline serum TSH levels, the obstetric history of the women, and the cause of the infertility in the couple, with a particular attention in case of female and idiopathic infertility.

## Abbreviations

ART: Assisted reproductive technology
ATA: American Thyroid Association
ICSI: Intra-cytoplasmic sperm injection
IVF: In-vitro fertilization
LT4: Levothyroxine
SCH: Subclinical hypothyroidism
TAI: Thyroid autoimmunity
Tg-abs: Thyroglobulin antibodies
TPO-abs: Thyroid peroxidase antibodies
